# Serum Ferritin Correlates With Liver Fat in Male Adolescents With Obesity

**DOI:** 10.3389/fendo.2020.00340

**Published:** 2020-06-18

**Authors:** Katharina Mörwald, Elmar Aigner, Peter Bergsten, Susanne M. Brunner, Anders Forslund, Joel Kullberg, Hakan Ahlström, Hannes Manell, Kirsten Roomp, Sebastian Schütz, Fanni Zsoldos, Wilfried Renner, Dieter Furthner, Katharina Maruszczak, Stephan Zandanell, Daniel Weghuber, Harald Mangge

**Affiliations:** ^1^Department of Pediatrics, University Hospital Salzburg, Paracelsus Medical University, Salzburg, Austria; ^2^Obesity Research Unit, University Hospital Salzburg, Paracelsus Medical University, Salzburg, Austria; ^3^First Department of Medicine, University Hospital Salzburg, Paracelsus Medical University, Salzburg, Austria; ^4^Department of Medical Cell Biology, University Uppsala, Uppsala, Sweden; ^5^Research Program for Receptor Biochemistry and Tumor Metabolism, Department of Pediatrics, University Hospital Salzburg, Paracelsus Medical University, Salzburg, Austria; ^6^Department of Women's and Children's Health, University Uppsala, Uppsala, Sweden; ^7^Department of Surgical Sciences, Radiology, University Uppsala, Uppsala, Sweden; ^8^Luxembourg Center for Systems Biomedicine, University Luxembourg, Luxembourg, Luxembourg; ^9^Department of Pediatrics and Adolescent Medicine, Salzkammergut-Klinikum, Vöcklabruck, Austria; ^10^Clinical Institute for Medical and Chemical Laboratory Diagnosis, Medical University Graz, Graz, Austria

**Keywords:** liver fat content, serum ferritin, pediatric obesity, non-alcocholic fatty liver disease, pediatric nonalcoholic fatty liver disease

## Abstract

Non-alcoholic fatty liver disease (NAFLD) contributes essentially to the burden of obesity and can start in childhood. NAFLD can progress to cirrhosis and hepatocellular carcinoma. The early phase of NAFLD is crucial because during this time the disease is fully reversible. Pediatric NAFLD shows unique features of histology and pathophysiology compared to adults. Changes in serum iron parameters are common in adult NAFLD and have been termed dysmetabolic iron overload syndrome characterized by increased serum ferritin levels and normal transferrin saturation; however, the associations of serum ferritin, inflammation, and liver fat content have been incompletely investigated in children. As magnetic resonance imaging (MRI) is an excellent measure for the degree of liver steatosis, we applied this method herein to clarify the interaction between ferritin and fatty liver in male adolescents. For this study, one hundred fifty male pediatric patients with obesity and who are overweight were included. We studied a subgroup of male patients with (*n* = 44) and without (*n* = 18) NAFLD in whom we determined liver fat content, visceral adipose tissue, and subcutaneous adipose tissue extent with a 1.5T MRI (Philips NL). All patients underwent a standardized oral glucose tolerance test. We measured uric acid, triglycerides, HDL-, LDL-, total cholesterol, liver transaminases, high sensitive CRP (hsCRP), interleukin-6, HbA1c, and insulin. In univariate analysis, ferritin was associated with MRI liver fat, visceral adipose tissue content, hsCRP, AST, ALT, and GGT, while transferrin and soluble transferrin receptor were not associated with ferritin. Multivariate analysis identified hsCRP and liver fat content as independent predictors of serum ferritin in the pediatric male patients. Our data indicate that serum ferritin in male adolescents with obesity is mainly determined by liver fat content and inflammation but not by body iron status.

## Introduction

Non-alcoholic fatty liver disease (NAFLD) is the most common liver disease in children and adolescents (1). The prevalence of pediatric NAFLD has swiftly increased paralleling the obesity epidemic. Estimations from epidemiological studies suggest that 7–10% of all and up to 30% of children with obesity are affected (2–4). It is dependent on age, ethnicity, and specific genetic determinants and is the highest in children with obesity with up to 38% (5, 6). The global prevalence of NAFLD was estimated to be 25.2% (95% CI: 22.10–28.65) with the highest prevalence in the Middle East and South America and the lowest in Africa. Metabolic comorbidities associated with NAFLD included obesity (51.34%; 95% CI: 41.38–61.20), type 2 diabetes (T2D, 22.51%; 95% CI: 17.92–27.89), hyperlipidemia (69.16%; 95% CI: 49.91–83.46%), hypertension (39.34%; 95% CI: 33.15–45.88), and metabolic syndrome (42.54%; 95% CI: 30.06–56.05) (7). Fibrosis progression proportion, and mean annual rate of progression in NASH were 40.76% (95% CI: 34.69–47.13) and 0.09 (95% CI: 0.06–0.12). Mechanisms leading to NAFLD include metabolic changes in glucose and lipid homeostasis, disturbed metabolic responses in context of a genetic predisposition, and an excess energy intake (8).

Serum ferritin is a reliable marker for body iron stores in the clinical routine. It is however, also strongly influenced by inflammatory stimuli, which significantly limits the use and interpretation of high serum ferritin concentrations. In the context of the metabolic syndrome and NAFLD, both iron stores and subclinical inflammation may therefore determine ferritin concentrations in any individual patient. Counteracting stimuli such as increased iron demand during growth and impaired iron uptake in obesity may further complicate the picture in a given patient (9). In adult studies, hyperferritinemia has been documented in obesity-related chronic inflammatory conditions such as type 2 diabetes mellitus, metabolic syndrome, and NAFLD. The histological evidence of hepatic iron accumulation has been reported to associate with hepatic fibrosis and advanced disease severity in adult patients with NAFLD, although, subsequent studies have not confirmed this association. Hence, results regarding the role of iron in human NAFLD remain inconclusive (10–12). Pediatric NAFLD shows unique features in liver histology and pathophysiology in comparison to adults (12, 13). In keeping with this, children with obesity and NAFLD exhibit no or only a rather mild intra-hepatic iron deposition. Furthermore, juveniles who are obese, frequently and typically have normal serum ferritin concentrations, hence the characteristic features of the dysmetabolic iron overload syndrome are usually not observed in children and adolescents (14–18). The association of ferritin concentrations with insulin resistance, abdominal obesity, and parameters of the metabolic syndrome and NAFLD have been extensively studied in adults (19) but only rarely in children (20–24). So far, the majority of previous pediatric studies applied ultrasound or liver enzymes as surrogates of NAFLD severity (10, 16). To date, there is a lack of studies accurately assessing the relationship between hepatic steatosis, associated markers of subclinical inflammation, and ferritin in pediatric patients. Although magnetic resonance imaging (MRI) has been shown to more reliably capture the degree of steatosis (25), only data from a South Korean study applying MRI in a pediatric age group exist so far (26).

Taken together the data of the role of ferritin as a potential marker of fatty liver disease in childhood are limited (27). The aim of this study was to assess how and to which extent ferritin is related to metabolic changes linked to obesity, in particular liver fat content.

## Materials and Methods

### Study Population, and Design

Between October 2012 and December 2016, we recruited 339 patients in an obesity specialist clinic in Salzburg (Austria) as part of the BETA JUDO study (BETA cell function in Juvenile Diabetes and Obesity, FP7-HEALTH-2011-two-stage, project number: 279153) in a cross-sectional study design. The inclusion criteria of patients were that they had to be in between the age of 10–18 years, overweight or obese according to the WHO criteria (BMI-SDS > 1). The exclusion criteria was a lack of consent or if the patients had any chronic liver disease. To this end, endocrine disorders (thyroid disease, diabetes type I), autoimmune, viral (viral hepatitis, HIV), or hereditary causes (Wilson disease, hereditary hemochromatosis, alpha-1 antitrypsin deficiency, celiac disease, lysosomal acid lipase deficiency) of liver disease, use of steatogenic drugs were excluded as confounders of elevated serum ferritin in all patients with increased serum transaminases (ALT, AST) >40 U/L. Patients did not report any alcohol intake. Full laboratory data sets of 150 male patients with obesity or are overweight were included out of which 62 male patients (35.4%) underwent additional MRI (liver fat content, abdominal visceral, and subcutaneous fat) and were selected for further investigations. Data of female patients (*n* = 164) were excluded due to potentially lower ferritin levels because of menorrhea.

Height and weight were assessed by means of a standardized, calibrated scale (Seca). BMI and BMI-SDS were calculated according to the WHO 2006–2007 reference population. Waist circumference (cm) and hip circumference (cm) were measured using a flexible tape. Blood pressure was measured twice, using a standardized clinical aneroid sphygmomanometer. Puberty staging was done according to Tanner and subjects were grouped as prepubertal (group 1 = Tanner I), pubertal (group 2 = Tanner II–IV), and postpubertal (group 3 = Tanner V). The ethical approvals for the study were obtained from the ethical committee of Salzburg (Number: 1544/2012). The study was carried out according to the Declaration of Helsinki. Written informed consent was achieved from all participants and at least one of their caregivers.

### Blood Sampling and Biochemical Analyses

Following an overnight fast, after blood sampling for all parameters but postprandial glucose, all patients underwent a standardized oral glucose tolerance test (OGTT, glucose dosage 1.75g/kg body weight) over 180 min as previously described (28, 29). OGTT was performed according to standard procedures by setting an intravenous line in an antecubital vein and subsequent blood draws were performed via this line at nine different time points after glucose challenge. Ferritin, soluble transferrin receptor, transferrin, iron, uric acid, triglycerides, HDL cholesterol, total cholesterol, and liver transaminases were measured using an enzymatic photometric test (Modular Analytics System, P-Modul, 917 by Roche Diagnostics). The evaluation of LDL cholesterol also required an enzymatic photometric test using Integra Manual by Roche Diagnostics. High sensitive CRP (hsCRP) was examined by an immunologic turbidimetric test (COBAS-Integra by Roche Diagnostics) and Interleukin 6 by an enzyme-linked immunosorbent assay (Modular Analytics System, E-Modul by Roche Diagnostics). HbA1c was measured by reversed-phase chromatography and lipoprotein (a) by a turbidimetric test (COBAS-Integra by Roche Diagnostics). Samples underwent immediate centrifugation at 2,500 g for 10 min at 4°C, subsequently aliquoted and frozen at −80°C. Plasma was consecutively used for the analysis of insulin in the central lab in Uppsala. Single-plex ELISA-kits for each analyte were used (Mercodia AB®, Uppsala, Sweden).

### Magnetic Resonance Imaging (MRI)

MRI-examinations were performed to determine liver fat content (LFC) and volumes of abdominal visceral adipose tissue (VAT) and subcutaneous adipose tissue (SAT) as previously described (29). All exams were performed using 1.5 T clinical MRI-systems from Philips Medical System (Netherlands) after a light meal and in close proximity to the OGTT. Water-fat imaging techniques were used throughout. The scans were done over 16 cm along the craniocaudal axis and centered on the L1 vertebra. The adipose tissue volumes were determined using a fully automated segmentation method that uses a filtering technique to separate VAT from SAT. Liver fat image reconstruction was done by a multi-resolution version of a method that employs a whole-image optimization approach (30). A single operator trained by an experienced radiologist performed the measurements by manual segmentation in the axial slices of the water images using the software ImageJ (version 1.42q, http://rsbweb.nih.gov/ij/).

### Definition of NAFLD

Patients with NAFLD had a liver fat content ≥5%, as measured by MRI. As previously described, a liver fat content cutoff of ≥5% and therefore a close relation between histopathological changes and liver fat fraction in MR-imaging have been promoted by various groups (31–36).

### Statistical Analysis

The data were analyzed descriptively showing results with mean and standard deviation for metric variables and number and percentages for categorical variables. Due to non-normality of the data, all group differences were assessed with non-parametric methods, namely Wilcoxon's test for unpaired samples. For assessing connections between variables, linear regression in univariate as well as multivariate case was applied. The coefficients are indicating the change of the dependent variable when the describing variable is changing by one unit. Additionally, standard errors and *p*-values are presented. All results are presented along with 95%-Confidence Intervals. Tests are performed at a significance level of 5%. *P*-values are corrected with Bonferroni method for multiple testing. In multiple regression, the variance inflation factor was used as an indicator of multicollinearity. All calculations were done with R (Version 3.6.0).

## Results

### General Group Characteristics

Table 1 shows descriptive data of the 150 male adolescents with obesity and are overweight. Mean age of juveniles with overweight/obesity was 13.02 ± 1.97 years and 80.7% were staged as pubertal. Mean BMI was 31.39 ± 6.73 kg/m^2^ and mean liver fat content as measured by MRI was 13.15 ± 12.30% (based on 62 subjects). Mean ferritin value was 57.33 ± 26.55 μg/L in the overweight/obese group.

**Table 1:** Descriptive data of all male patients, with and without NAFLD as determined by MRI.

	**Overweight/obese (*n* = 150)**	**NAFLD (*n* = 44)**	**Non-NAFLD (*n* = 18)**	***p***
Age (years)	13.02 ± 1.97	13.65 ± 2.10	13.51 ± 1.68	n.s.
Tanner stage^*^	I: 20 (13.3%)	I: 5 (11.4%)	I: 1 (5.6%)	
	II–IV: 121 (80.7%)	II–IV: 36 (81.8%)	II–IV: 17 (94.4%)	
	V: 8 (0.5%)	V: 3 (7.8%)	V: 0 (0 %)	
BMI (kg/m^2^)	31.39 ± 6.73	33.01 ± 5.38	28.98 ± 4.56	0.007
BMI-SDS	2.91 ± 0.57	3.02 ± 0.53	2.51 ± 0.53	0.002
SBMI (kg/m^2^)	35.23 ± 3.96	36.05 ± 3.62	32.74 ± 3.21	0.002
Waist circumference (cm)	102.59 ± 15.54	107.14 ± 12.68	98.33 ± 14.66	0.024
Hip circumference (cm)	105.22 ± 14.17	107.67 ± 12.17	105.19 ± 12.57	n.s.
Waist-to-hip-ratio	0.98 ± 0.07	1.00 ± 0.07	0.94 ± 0.08	0.005
Total body fat (%)	37.61 ± 7.75	39.63 ± 7.79	31.98 ± 7.25	0.001
MRI VAT volume (cm^3^)	1584.03 ± 593.40	1721.62 ± 626.22	1237.57 ± 345.86	0.002
MRI SAT volume (cm^3^)	6271.76 ± 2386.84	6762.69 ± 2200.47	5213.06 ± 2447.39	0.010
MRI liver fat content (%)	13.15 ± 12.30	17.28 ± 12.42	3.05 ± 0.89	0.000
RR systolic (mmHg)	122.04 ± 13.93	124.22 ± 13.40	120.86 ± 11.54	n.s.
RR diastolic (mmHg)	65.52 ± 10.82	7.02 ± 11.24	63.06 ± 5.85	n.s.
HbA1c (mmol/mol)	35.40 ± 3.48	35.72 ± 4.48	35.39 ± 2.06	n.s.
Total cholesterol (mmol/L)	4.03 ± 0.87	4.18 ± 0.96	4.05 ± 0.74	n.s.
LDL cholesterol (mmol/L)	2.20 ± 0.73	2.39 ± 0.86	2.12 ± 0.54	n.s.
HDL cholesterol (mmol/L)	1.35 ± 0.34	1.24 ± 0.21	1.50 ± 0.51	n.s.
Triglycerides (mmol/L)	1.09 ± 0.61	1.22 ± 0.65	0.94 ± 0.54	n.s.
AST (μkat/L)	0.53 ± 0.24	0.60 ± 0.36	0.47 ± 0.10	n.s.
ALT (μkat/L)	0.61 ± 0.49	0.82 ± 0.75	0.44 ± 0.19	0.012
GGT (μkat/L)	0.40 ± 0.28	0.48 ± 0.40	0.31 ± 0.13	0.024
Uric acid (μmol/L)	370.62 ± 127.61	362.17 ± 92.00	382.69 ± 86.03	n.s.
hsCRP (mg/L)	4.09 ± 4.48	4.74 ± 5.19	2.96 ± 3.40	n.s.
IL-6 (pg/mL)	7.34 ± 2.01	7.75 ± 3.36	7.03 ± 0.12	n.s.
TNF alpha (pg/mL)	8.59 ± 2.15	8.70 ± 1.46	8.21 ± 1.97	n.s.
STFR (mg/L)	4.49 ± 1.66	4.88 ± 2.17	4.01 ± 1.17	n.s.
Iron (μg/dL)	41.55 ± 20.18	39.90 ± 18.64	42.92 ± 17.37	n.s.
TF (mg/dL)	270.11 ± 35.79	271.52 ± 40.76	287.00 ± 46.87	n.s.
Ferritin (μg/L)	57.33 ± 26.55	59.82 ± 29.17	44.35 ± 28.76	0.025
OGTT fasting glucose (mmol/L)	4.77 ± 0.54	4.73 ± 0.59	4.89 ± 0.62	n.s.
OGTT 120 min. glucose (mmol/L)	6.49 ± 1.42	6.58 ± 1.52	6.02 ± 1.37	n.s.
OGTT fasting insulin (μIU/mL)	18.44 ± 11.09	21.48 ± 11.77	14.21 ± 6.91	n.s.

*Data are expressed as mean ± standard deviation*.

*CI, confidence interval; LB, lower bound; UB, upper bound; SD, standard deviation; MRI/ Tanner staging/ PNPLA3 was only performed in overweight/ obese patients but not in controls due to decision of the ethical board; BMI, body mass index; BMI-SDS, body mass index standard deviation score; SBMI, smart BMI; RR, blood pressure; HbA1c, hemoglobin A1c; LDL, low density lipoprotein; HDL, high density lipoprotein; AST, aspartate aminotransferase; ALT, alanine aminotransferase; GGT, gamma glutamyl transferase; hsCRP, high sensitivity C-reactive protein; IL-6, interleukin 6; TNF, tumor necrosis factor; STFR, soluble transferring receptor; TF, transferrin; OGTT, oral glucose tolerance test; MRI, magnetic resonance imaging; VAT, visceral adipose tissue; SAT, subcutaneous adipose tissue*.

* *Tanner staging I–V: I, prepubertal; II–IV, pubertal; V = postpubertal*.

### NAFLD Group Characteristics

Descriptive data of 44 male adolescents with NAFLD and 18 without NAFLD (defined by liver fat content ≥5 and <5%, respectively) are shown in Table 1. In Table 2, descriptive data of the 62 male NAFLD patients are presented and categorized into quartiles of liver fat content, resulting in the following cut-offs: 25% = 3.9% liver fat content, 50% = 6.6% liver fat content, 75% = 20.4% liver fat content. Data are means with standard deviations, and *p*-values for the highest quartile in differentiation to the remaining three quartiles. Mean age and pubertal staging are similar between quartiles. BMI-SDS and SBMI is significantly higher in the highest quartile compared to the other three. In summary, characteristics of the metabolic syndrome showed a “metabolically unhealthier” phenotype in quartile 4, although only 2-h-glucose level in an oral glucose tolerance test was significantly different between the highest quartiles and the other remaining quartiles. Liver transaminases rise alongside to quartiles and show significantly higher levels in quartile 4. Inflammatory parameters show higher levels in quartile 4, without reaching a significant difference. Measured by MRI, liver fat, and visceral adipose tissue content are significantly elevated in quartile 4.

**Table 2:** Descriptive data of **male** NAFLD patients (defined according to liver fat content ≥ 5%, *n* = 62) categorized into quartiles of liver fat content (cut-offs: 25% = 3.8925%, 50% = 6.62%, 75% = 20.435%).

	**Quartile 1**	**Quartile 2**	**Quartile 3**	**Quartile 4**
Age (years)	13.41 ± 1.73	14.28 ± 1.53	13.61 ± 2.21	13.16 ± 2.34
Tanner stage^*^	I: 1 (0.2%)	I: 0	I: 1 (0.2%)	I: 4 (0.7%)
	II–IV: 15 (24.2%)	II–IV: 14 (22.6%)	II–IV: 14 (22.6%)	II–IV: 10 (16.1%)
	V: 0	V: 1 (0.2%)	V: 0	V: 2 (0.3%)
BMI (kg/m^2^)	29.01 ± 4.85	32.26 ± 5.32	32.56 ± 5.13	33.62 ± 5.13
BMI-SDS	2.52 ± 0.56	2.83 ± 0.51	2.97 ± 0.48	**3.18** **±** 0.58^*^
SBMI (kg/m^2^)	32.83 ± 3.39	34.76 ± 3.44	35.62 ± 3.28	**37.17** **±** 3.95^*^
Waist circumference (cm)	98.19 ± 15.21	107.47 ± 13.77	105.53 ± 10.68	107.38 ± 13.96
Hip circumference (cm)	105.28 ± 13.32	109.20 ± 11.01	106.80 ± 11.05	106.66 ± 14.02
Waist-to-hip-ratio	0.93 ± 0.08	0.98 ± 0.07	0.99 ± 0.06	1.01 ± 0.07
Total body fat (%)	31.36 ± 6.74	39.22 ± 7.04	38.78 ± 8.85	40.42 ± 7.92
MRI VAT volume (cm^3^)	1220.41 ± 366.06	1447.64 ± 417.38	1660.52 ± 476.99	**1991.33** **±** 780.82^**^
MRI SAT volume (cm^3^)	5159.33 ± 2577.45	6942.97 ± 2426.13	6480.27 ± 1884.64	6715.11 ± 2301.43
MRI liver fat content (%)	2.90 ± 0.81	5.52 ± 0.64	12.47.4.35	**31.20** **±** 8.86^***^
RR systolic (mmHg)	120.78 ± 12.28	122.03 ± 10.20	124.2 ± 13.71	125.94 ± 15.34
RR diastolic (mmHg)	62.75 ± 6.13	67.70 ± 8.35	67.00 ± 12.74	66.22 ± 12.04
HbA1c (mmol/mol)	35.19 ± 2.07	36.07 ± 1.94	34.40 ± 3.62	36.81 ± 6.16
Total cholesterol (mmol/L)	4.09 ± 0.77	4.24 ± 0.95	4.10 ± 0.73	4.15 ± 1.16
LDL cholesterol (mmol/L)	2.12 ± 0.58	2.50 ± 0.84	2.33 ± 0.64	2.30 ± 1.03
HDL cholesterol (mmol/L)	1.52 ± 0.52	1.27 ± 0.24	1.19 ± 0.18	1.26 ± 0.25
Triglycerides (mmol/L)	0.97 ± 0.56	1.02 ± 0.46	1.24 ± 0.62	1.32 ± 0.81
AST (μkat/L)	0.46 ± 0.10	0.44 ± 0.10	0.49 ± 0.13	**0.84** **±** 0.50^***^
ALT (μkat/L)	0.44 ± 0.20	0.39 ± 0.08	0.57 ± 0.29	**1.41** **±** 0.97^***^
GGT (μkat/L)	0.30 ± 0.14	0.32 ± 0.11	0.36 ± 0.12	**0.72** **±** 0.58^***^
Uric acid (μmol/L)	381.08 ± 89.09	363.65 ± 100.14	348.84 ± 74.03	376.61 ± 98.87
hsCRP (mg/L)	3.11 ± 3.58	4.03 ± 3.73	3.76 ± 5.14	5.96 ± 6.16
IL-6 (pg/mL)	7.03 ± 0.13	7.83 ± 3.20	7.00 ± 0.00	8.28 ± 4.69
TNF alpha (pg/mL)	8.17 ± 1.86	8.54 ± 1.64	8.40 ± 1.46	9.11 ± 1.49
STFR (mg/L)	3.87 ± 1.12	4.12 ± 0.79	5.99 ± 3.30	4.57 ± 0.83
Iron (μg/dL)	43.55 ± 18.07	40.73 ± 16.78	37.45 ± 18.65	41.30 ± 21.20
TF (mg/dL)	283.64 ± 47.61	260.55 ± 51.35	291.36 ± 32.64	267.00 ± 32.85
Ferritin (μg/L)	46.52 ± 29.10	47.86 ± 16.49	55.83 ± 35.53	**73.44** **±** 30.06^*^
OGTT fasting glucose (mmol/L)	4.79 ± 0.59	4.71 ± 0.66	4.72 ± 0.58	4.87 ± 0.61
OGTT 120 min. glucose (mmol/L)	5.96 ± 1.37	5.88 ± 1.14	6.38 ± 0.99	**7.42** **±** 1.87^**^
OGTT fasting insulin (μIU/mL)	13.58 ± 6.94	16.88 ± 8.83	20.63 ± 7.32	27.94 ± 15.83

*Data are expressed as mean ± standard deviation*.

P-values are calculated between quartile 4 and quartile 1-3 and presented as follows:

* p < 0.05,

** p < 0.01,

*** *p < 0.001*.

*NAFLD, patients with non-alcoholic fatty liver disease; BMI, body mass index; BMI-SDS, body mass index standard deviation score; SBMI, smart BMI; RR, blood pressure; HbA1c, hemoglobin A1c; LDL, low density lipoprotein; HDL, high density lipoprotein; AST, aspartate aminotransferase; ALT, alanine aminotransferase; GGT, gamma glutamyl transferase; hsCRP, high sensitivity C-reactive protein; IL-6, interleukin 6; TNF, tumor necrosis factor; STFR, soluble transferring receptor; TF, transferrin; OGTT, oral glucose tolerance test; MRI, magnetic resonance imaging; VAT, visceral adipose tissue; SAT, subcutaneous adipose tissue*.

* *Tanner staging I–V: I, prepubertal; II–IV, pubertal; V, postpubertal*.

### Ferritin as Cardiometabolic Risk Factor Independent of Iron Overload

Concerning iron metabolism, ferritin was significantly higher between NAFLD and non-NAFLD groups and in quartile 4 compared to the lower quartiles. In contrast, standard indicators of iron metabolism like soluble transferrin receptor, serum iron, and transferrin were similar between groups. In univariate analysis (Table 3), ferritin was directly correlated with MRI liver fat and visceral adipose tissue content as well as hsCRP, AST, ALT, and GGT. In multivariate analysis (Table 4), we tested which parameter best predicted serum ferritin in male obese and overweight juveniles. We included parameters with a significant correlation to ferritin from the univariate analysis into the multivariate model. Further, with an adjusted *R*-squared as criterion, none of the other covariates described any more of the variance in ferritin (data not shown). ALT, AST, and GGT were excluded from multivariate analysis due to multicollinearity. For multivariate analysis, we therefore chose hsCRP, MRI liver fat content, and MRI visceral adipose tissue content as variables, of which hsCRP and liver fat content independently predicted ferritin. Of note, liver fat content was positively correlated with visceral adipose tissue (coefficient 0.36). However, there was no indication of multicollinearity (variance inflation factor, data not shown).

**Table 3:** Univariate analyses in **male** overweight/ obese patients (*n* = 85^#^).

**Ferritin vs. age**	**Coefficient**	***t*-value**	***p*-value**	**CI LB**	**CI UB**
	**0.428**	**1.381**	**0.757**	**−2.319**	**3.175**
BMI	0.376	1.084	0.282	−0.314	1.065
BMI-SDS	5.600	1.248	0.216	−3.330	14.529
Waist circumference	0.216	1.386	0.170	−0.094	0.525
Systolic blood pressure	0.104	0.516	0.607	−0.296	0.503
MRI liver fat content	0.942	3.150	**0.003**	0.337	1.546
MRI subcutaneous adipose tissue	0.001	0.667	0.509	−0.002	0.004
MRI visceral adipose tissue	0.013	2.023	**0.0496**	0.000	0.025
120 minutes glucose	3.851	1.823	0.072	−0.351	8.052
Triglycerides	0.055	0.981	0.330	−0.056	0.166
HDL-cholesterol	−0.029	−0.142	0.888	−0.443	0.384
Fasting insulin	0.026	0.667	0.507	−0.052	0.104
hs-CrP	1.269	2.502	**0.014**	0.260	2.278
IL-6	1.293	1.255	0.213	−0.757	3.343
TNF-alpha	2.252	1.554	0.124	−0.631	5.134
AST	28.345	3.050	**0.003**	9.864	46.844
ALT	18.116	4.054	**0.000**	9.226	27.006
GGT	35.142	4.237	**0.000**	18.639	51.645

*CI LB, confidence interval lower bound; CI UB, confidence interval upper bound; ALT, alanine aminotransferase; AST, aspartate aminotransferase; GGT, gamma glutamyl transferase; BMI, body mass index; BMI-SDS, body mass index standard deviation score; MRI, magnetic resonance imaging; HDL, high density lipoprotein; hsCRP, high sensitivity C-reactive protein; IL-6, interleukin 6; TNF, tumor necrosis factor*.

# *n = 84 for BMI, BMI-SDS, waist circumference, triglycerides, HDL-cholesterol, hs-CrP, IL-6, TNF-alpha; n = 82 for systolic blood pressure; n = 42 for liver fat content; n = 43 for visceral and subcutaneous adipose tissue; n = 83 for 120 min glucose; n = 74 for fasting insulin*.

**Table 4:** Multivariate regression analysis: predictors of ferritin in a male overweight/obese pediatric cohort (*n* = 42).

	**Coefficients**	**Standard error**	***t*-value**	***p*-value**
Intercept	31.259	9.686	3.227	0.003
hs-CRP (mg/L)	1.554	0.697	2.230	0.032^*^
MRI liver fat content (%)	0.667	0.313	2.125	0.040^*^
MRI visceral adipose tissue (%)	0.004	0.006	0.647	0.522

*hs-CrP, high sensitivity C-reactive protein; MRI, magnetic resonance imaging*.

* *p < 0.05*.

## Discussion

Obesity is increasing worldwide (37) and young people are particularly affected. This is alarming because young obese people will suffer from serious diseases later in life such as myocardial infarction, stroke, or cancer. As people become increasingly older, it is vital to remain in a healthy condition as you age (37). Liver disease contributes essentially to the pathologic burden of obesity and can start as early as in childhood. Fat accumulation in the liver is a hallmark of non-alcoholic fatty liver disease (NAFLD) which rapidly increases worldwide (38). NAFLD can progress from steatosis (NAFL) to steatohepatitis (NASH), cirrhosis, and hepatocellular carcinoma (HCC).

Approximately one-third of adult patients with NAFLD have a disturbed iron homeostasis but iron excess is to the best of our knowledge absent in pediatric NAFLD. Systemic iron overload associates with elevated serum ferritin and normal or mildly elevated transferrin saturation. The histological iron deposition pattern in these patients shows iron deposition in both hepatocytes and liver macrophages or only one of these cells, and has been named dysmetabolic iron overload syndrome (DIOS). Negative long-term effects of the hepatic iron deposition on the clinical course of NAFLD may occur because iron catalyzes the formation of toxic hydroxyl radicals, which mediate cellular damage potentially augmenting fibrosis progression in NAFLD (39). Conversely, reduction of body iron can restore insulin sensitivity, and epidemiological evidence suggests that it delays the onset of complications such as type 2 diabetes mellitus, cardiovascular disease, and advanced liver disease. Iron accumulation in adult NAFLD is due to inhibition of iron mobilization from hepatocytes and Kupffer cells. The impaired iron export relates to inflammation and metabolic derangements and involves key iron regulators such as hepcidin and ferroportin (40). We therefore aimed to elucidate whether serum ferritin links to indicators of body iron status or metabolic parameters in a well-characterized obese male children cohort. We observed that liver fat content determined by MRI and hsCRP were the best predictors of serum ferritin in male pediatric patients with obesity. These results show that already in childhood and adolescence serum ferritin increases after metabolic derangements with special importance of fat storage in the liver and reactive metabolic inflammation. Despite increased ferritin, the main indicators of increasing body iron stores and erythropoietic iron demand like soluble transferrin receptor, serum iron, and transferrin remained unchanged over quartiles. Adolescence is an important period of nutritional vulnerability and iron needs are elevated because of intensive growth and muscular development. However, this increased iron use might protect metabolically unhealthy young males from the development of actual iron overload (41). This further strengthens the suggestion that serum ferritin may be rather linked to metabolic inflammation (40) and not to a dysmetabolic iron overload syndrome which although may develop later in life in these subjects.

Moreover, the identification of potential biomarkers of the NAFLD and metabolic syndrome are an important clinical agenda. Hepatic fat content is an important estimate of systemic metabolic health. Our study revealed that MRI-assessed liver fat content and hsCRP best predicted serum ferritin values. These results suggest that ferritin may serve as a marker of early fatty liver disease in childhood obesity, at least in males. This is of interest, as non-invasive surrogate scores such as the fatty liver index have been shown to poorly predict liver fat content in children with obesity (42).

### Limitation

The cross-sectional study design does not provide reliable information about causal relationships. Possibly, ferritin may be elevated because of accumulation of liver fat or ferritin may itself contribute causally to liver fat accumulation. Moreover, in more severe NAFLD, low degree of hepatic steatosis can be due to replacement of liver fat by fibrotic tissue (43). It is not possible to draw conclusions on underlying liver disease severity from our data. By assessing liver fat content by MRI in our patients, we could not distinguish steatosis and fibrosis. Ultimately, NAFLD/NASH is a diagnosis of exclusion, and liver biopsy is required to confirm the diagnosis, stage the disease, rule out other liver diseases, and determine the need for and urgency of aggressive therapy. In addition, due to missing values and thus reduced statistical power the results of the regression analysis must be interpreted cautiously. A further inherent limitation of ferritin as a marker of metabolic changes is that it is solely applicable in males due to the overwhelming impact of menstrual blood loss on iron parameters in female adolescents.

## Conclusion

We showed herein that serum ferritin concentrations in male adolescents are related to hepatic lipid accumulation as verified by MRI and hsCRP as a marker of concomitant metabolic inflammation. These findings suggest that inflammation is already present in early phases of fatty liver disease and that elevated ferritin may link more closely to the development of adverse metabolic and inflammatory consequences of overweight and obesity than to iron status. Our results add novel information to the interpretation of serum ferritin concentrations in male adolescents with obesity as serum ferritin appears to increase in serum more in relation to liver fat and inflammation than to body iron stores. The clinical use of serum ferritin as a marker for NAFLD in children remains to be elucidated in future investigations including the evaluation of liver disease severity.

## Data Availability Statement

The datasets generated for this study are available on request to the corresponding author.

## Ethics Statement

The studies involving human participants were reviewed and approved by Ethics commitee Salzburg, Austria. Written informed consent to participate in this study was provided by the participants' legal guardian/next of kin.

## Author Contributions

KMö and SB was involved in data acquisition and manuscript preparation. HA, AF, JK, HMane, and FZ was involved in data acquisition. EA and WR was involved in manuscript preparation. PB was involved in data acquisition and headed the Beta-JUDO study. KR was involved in data acquisition and storage. DW and HMang were both involved in manuscript preparation and data acquisition. All authors contributed to the article and approved the submitted version. DF, KMa, and SZ contributed to interpretation of data and manuscript revision.

## Conflict of Interest

DW has received consultant fees from Novo Nordisk. HA and JK are cofounders, stock owners, and employees of Antaros Medical, Mölndal, Sweden. The remaining authors declare that the research was conducted in the absence of any commercial or financial relationships that could be construed as a potential conflict of interest.
